# A Shorted Stub Loaded UWB Flexible Antenna for Small IoT Devices

**DOI:** 10.3390/s23020748

**Published:** 2023-01-09

**Authors:** Esraa Mousa Ali, Wahaj Abbas Awan, Mohammed S. Alizaidi, Abdullah Alzahrani, Dalia H. Elkamchouchi, Francisco Falcone, Sherif S. M. Ghoneim

**Affiliations:** 1 Faculty of Aviation Sciences, Amman Arab University, Amman 11953, Jordan; 2 Department of Information and Communication Engineering, Chungbuk National University, Cheongju 28644, Republic of Korea; 3 Department of Electrical Engineering, College of Engineering Taif University, P.O. Box 11099, Taif 21944, Saudi Arabia; 4 Department of Information Technology, College of Computer and Information Sciences, Princess Nourah bint Abdulrahman University, P.O. Box 84428, Riyadh 11671, Saudi Arabia; 5 Electrical Engineering and Communications Department, Universidad Pública de Navarra, Campus Arrosadía, E-31006 Pamplona, Spain; 6 Institute of Smart Cities, Universidad Pública de Navarra, Campus Arrosadía, E-31006 Pamplona, Spain; 7 Tecnologico de Monterrey, School of Engineering and Sciences, Monterrey 64849, Mexico

**Keywords:** compact devices, UWB antenna, flexible electronics

## Abstract

In this manuscript, a compact in size yet geometrically simple Ultra-Wideband (UWB) antenna is demonstrated. The flexible-by-nature substrate ROGERS 5880, having a thickness of 0.254 mm, is utilized to design the proposed work. The antenna configuration is an excerpt of a traditional rectangular monopole antenna resonating at 5 GHz. Initially, a pair of triangular slots are employed to extend the impedance bandwidth of the antenna. In addition, a semi-circular-shaped, short-ended stub is connected at the upper edges of the patch to further increase the operational bandwidth. After optimization, the proposed antenna offers UWB ranging from 2.73–9.68 GHz, covering almost the entire spectrum allocated globally for UWB applications. Further, the antenna offers a compact size of 15 × 20 mm^2^ that can easily be integrated into small, flexible electronics. The flexibility analysis is done by bending the antenna on both the *x* and *y* axes. The antenna offers performance stability in terms of return loss, radiation pattern, and gain for both conformal and non-conformal conditions. Furthermore, the strong comparison between simulated and measured results for both rigid and bent cases of the antenna, along with the performance comparison with the state-of-the-art, makes it a potential candidate for present and future compact-sized flexible devices.

## 1. Introduction

The UWB (ultra-wideband) radio demonstrates features such as broad bandwidth, low power spectrum levels, high data-rate transmission, good phase linearity, and auspicious radiation performance [1]. As a result, the UWB technology has promising prospects in diverse applications, such as the Internet of Things (IoT), surveillance systems, wireless sensor networks, and medical applications [2,3]. Since the year 2002, researchers have intensified their efforts in this area after the allocation of the frequency range 3.1–10.6 GHz for unlicensed UWB indoor wireless communication by the United States Federal Communication Commission (FCC) [4]. Whereas, the Electronic Communication Committee (ECC) has designated the 6–8.5 GHz band spectrum for UWB applications [5].

In addition, with the progression of wireless communication standards, an enormous number of devices and gadgets are required to be connected to the internet. The assortment of interconnected devices accelerated the idea of designing flexible and robust antennas capable of being mounted on curved or wearable devices [6,7,8]. Moreover, various conductive and substrate materials have been investigated to be employed as flexible substrates, such as Kapton, paper, polyethylene terephthalate (PET), polydimethylsiloxane (PDMS), polyamide, and textile fabric [9,10,11,12,13]. Flexible antennas are desired to have acceptable radiation characteristics in conformal and rigid configurations.

Recently, numerous approaches have been reported in the literature for attaining the UWB characteristics [5,6,7] and [14,15,16]. However, very few UWB antennas have been presented with flexible substrates [17,18,19,20,21,22]. For instance, the reported work in [17] is made up of a co-planar waveguide (CPW)-fed circular monopole antenna designed using a liquid crystalline polymer (LCP)-based substrate. The antenna offers a wideband of 2.5–11 GHz along with an average gain of 2 dB and an overall size of 40 × 22 × 0.1 mm^3^. On the other hand, an inkjet-printed flexible Multiple-Input, Multiple-Output (MIMO) antenna is presented in [18]. It has a relatively compact size of 22 × 31 × 0.125 mm^3^ and offers a wide bandwidth ranging from 3.43–10.1 GHz, accompanied by an average gain of 1.7 dBi. Similarly, in [19], a flexible antenna having physical dimensions of 38 × 22 × 0.1 mm^3^ is proposed for wearable applications. Although the antenna offers a wideband of 2.8 GHz (5.8–8.6 GHz), this work cannot be used for UWB applications requiring bandwidth ranging from 3.1 to 10 GHz. Another intriguing report conducted in [20] describes a photo paper-based inkjet-printed antenna for IoT applications. The antennas offer a wideband over 3.2–15 GHz, having an average gain of 4.87 dB along with an overall dimension of 33.1 × 32.7 × 0.254 mm^3^.

Further, in [21], a flexible antenna is designed on a polyimide substrate for UWB applications. The polyimide substrate-based antenna is designed after the surface modification by utilizing an in situ self-metallization technique. The antenna offers an ultra-wide bandwidth of 1.35–16.4 GHz as well as a high realized gain of >2.8 dB in the operational region. Lastly, a nanocomposite material-based organic antenna is presented for UWB and flexible applications [22]. The antenna comprises 48 × 34.9 × 0.13 mm^3^ and has an operational impedance bandwidth of 2–7 GHz, along with mismatched performance for unbent and bent conditions. Thus, it can be concluded from the aforementioned decision that the UWB antennas proposed in [17,18,19,20,21,22] exhibit larger dimensions. Furthermore, the reported work in [21,22] involves costly substrates and complex fabrication methodologies. As the state-of-the-art systems require compact antennas, it would therefore be advantageous to employ a more compact UWB antenna with conformal capabilities for diverse services to comply with the commercial and functional requirements.

Therefore, this work offers the design of a miniaturized UWB antenna realized on a flexible substrate. The overall substrate dimensions of the proposed design are 20 × 15 × 0.254 mm^3^_,_ which is relatively compact compared to the state-of-the-art. The proposed structure exhibits adequate radiation characteristics for both rigid and conformal arrangements. Therefore, the suitability of the proposed antenna is ascertained for both rigid and conformal electronic devices. The forthcoming parts of the paper are split as follows: Section 2 describes the methodology used to design the proposed antenna, while Section 3 discusses the performance parameters of the proposed antenna. Finally, Section 4 concludes the manuscript, which is accompanied by references.

## 2. Methodology of the Proposed Antenna and Methodology

### 2.1. UWB Antenna

**Figure 1:**
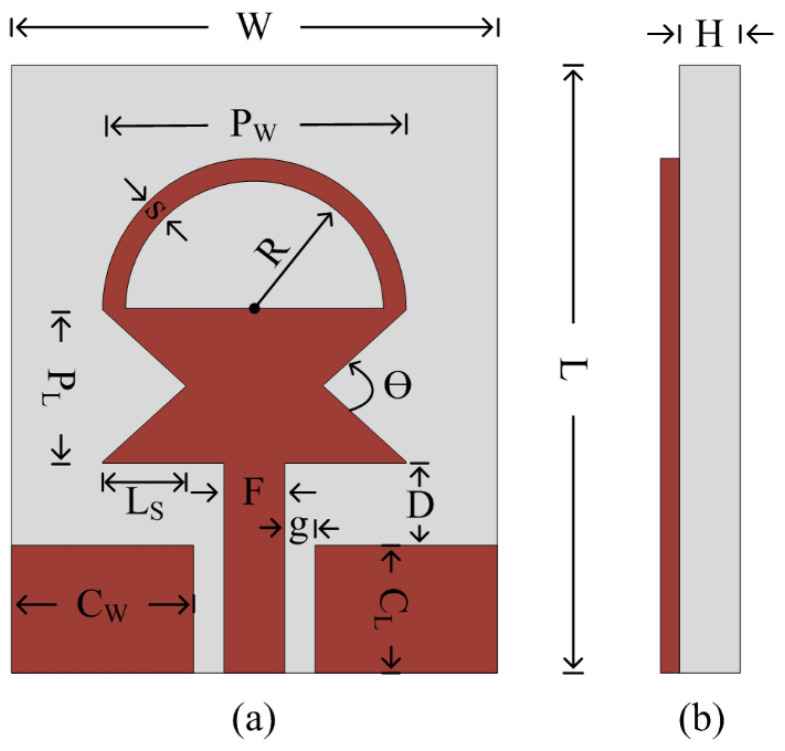
Geometry of the proposed antenna: (**a**) front view; (**b**) side view L = 20 mm; W = 15 mm; C_L_ = 5.5 mm; C_W_ = 6 mm; g = 0.5 mm; F = 2 mm; D = 2 mm; H = 0.254 mm; P_L_ = 5 mm; P_w_ = 13 mm; L_s_ = 2.5 mm; R = 4.5 mm; s = 1.5 mm; θ = 90°.

Figure 1 depicts the layout of the proposed antenna. The radiating structure is placed at the top of the flexible material ROGERS 5880, which has an electric permittivity of 2.2 along with a loss tangent of 0.0009. The radiator is fed using the CPW feeding technique, owing to the advantage of its uniplanar structure, which increases its potential for use with electronic circuits [23]. Furthermore, because of the similar structure, CPW feed aids in achieving a compact antenna size as well as ease of fabrication [24]. The antenna radiator consists of a rectangular-shaped quarter-wave monopole whose both sides are truncated using triangular-shaped slots. Afterwards, a semi-circular-shaped short stub is inserted at the top of the radiator, which helps in achieving an ultra-wideband. The working principle of the antenna is explained in the forthcoming section, along with the results of various design techniques utilized to achieve the proposed UWB antenna.

### 2.2. Antenna Design Methodology

**Figure 2:**
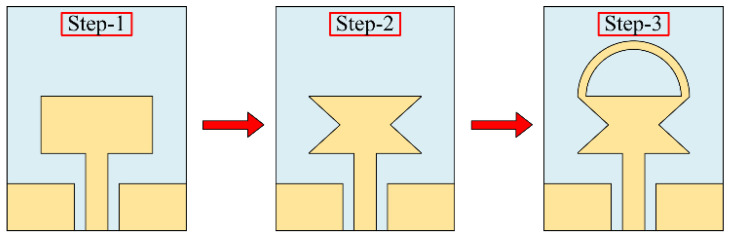
Different antennas are utilized to achieve the proposed UWB antenna design.

Figure 2 portrays the geometrical structure of various antennas utilized to design the proposed work. The design methodology consists of three major steps.

*Step-1:* The design of a compacts size conventional quarter-wave monopole antenna.

*Step-2:* Band enhancement of the quarter-wave monopole antenna by truncating the sides of the radiator.

*Step-3:* Further operational band enhancement of the truncated monopole antenna by loading the short-ended stub. Initially, a CPW-fed, rectangular-haped quarter-wave monopole antenna is designed. The length (*P_L_*) of the radiator for any desired resonance (*f*_0_) can be estimated by utilizing Equations (1) and (2), as proven in [25]:

(1) $$P_{L}=\frac{c}{4f0\sqrt{\epsilon_{eff}}}$$

Here *ϵ_eff_* is the effective permittivity of the substrate, which is given as:

(2) $$\epsilon_{eff}\approx \frac{\epsilon r+1}{2}+\frac{\epsilon r+1}{2}{\left(1+12\left(\frac{P_{w}}{H}\right)\right)}^{-0.5}$$

Here, *ϵ_r_* is the permittivity of the substrate, *P_W_* is the width of the radiator, and H is the thickness of the substrate.

Besides, for broadband antenna, the lower cut-off frequency (*F_L_*) of the monopole antenna can be estimated by the following expression provided in [26]:

(3) $$F_{l}=\frac{7.2\;}{\left(l+r+p\right)\times k}$$

for proposed antenna, the parameters in the above equations are:

$$l=\frac{P_{w}}{2}\;r=\frac{P_{L}}{4\pi }\;p=D\;k=\sqrt{\epsilon_{eff}}$$

After putting the parameters into Equation (3), the equations become:

(4) $$F_{l}=\frac{7.2\;}{\left(\frac{P_{w}}{2}+\frac{P_{L}}{4\pi }+D\right)\times \sqrt{\epsilon_{eff}}}$$

**Figure 3:**
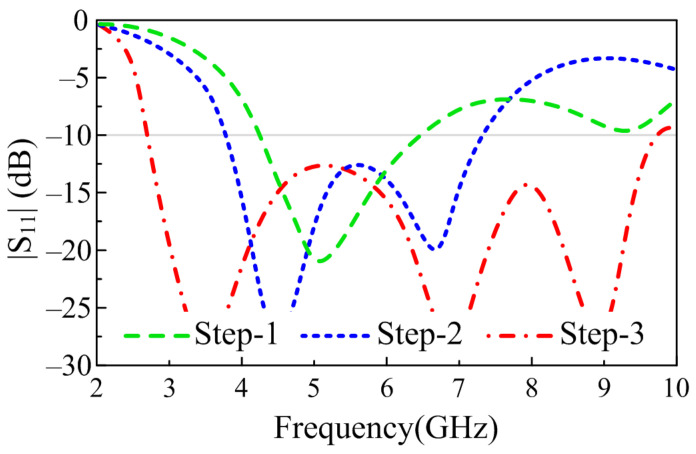
|S_11_| of the various antenna involve in designing of proposed work.

Equation (4) gives the lower cut-off frequency of 4.19 GHz, which is remarkably close to the simulated results having a lower cut-off frequency of 4.23 GHz, as depicted in Figure 3. Furthermore, Equation (4) also shows that the lower cut-off frequency of a monopole antenna can be adjusted by varying the length and width of the patch along with the space between the radiator and CPW ground, denoted by D.

However, due to the size limitation of the antenna, further variation was not possible, and the optimized antenna offers a broadband of 4.23–6.64 GHz, as illustrated in Figure 3. In order to broaden the bandwidth of the radiator even further, truncating the corners of the radiator is utilized. Various techniques, including etching slots [27], inserting vias [28], and loading open-ended stubs [29], are widely studied to achieve wide bandwidth. However, etching slots requires a bigger radiator, inserting vias requires a ground plane at the back of the radiator, and open-ended stubs require more space. Therefore, instead of utilizing conventional techniques, the truncated corner technique is exploited. Furthermore, rather than truncating the corners of the radiator as utilized in [8,10,28], the sides of the radiator are truncated by using a triangular-shaped structure, as depicted in Figure 2. The variation in the shape of the radiator due to the etched slot causes the flow of current to redistribute itself across the surface of the radiator, and with optimization, it results in the generation of a wide band ranging from 3.8–7.35 GHz, as depicted in Figure 3.

In a decisive step, a semi-circular-shaped stub was added at the top-corner of the radiator. The loading of the stub introduces additional impedance that can be approximated by the following relationship [30]:

*Z*_*SC*_ = *j*
*Z*_*O*_ tan (*β*l) (5)

Here, *Z_O_* is the input impedance of 50 Ω, *β* is the per-unit change in length and can be computed as *β = 2π/λ*, l is the physical length of the stub and can be computed by using the arc length (*L_A_*) formula of *L_A_* = *π/180 × θ × R*, and *j* is the unit imaginary number.

**Figure 4:**
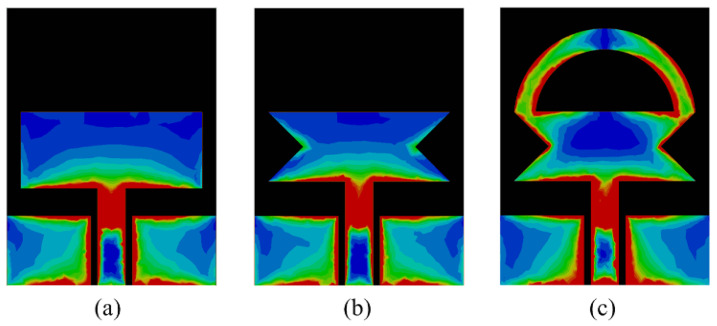
Surface current distribution at 3.5 GHz (**a**) step-1 (**b**) step-2 (**c**) step-3.

The addition of extra impedance due to the insertion of the stub provides a good match with the transmission line and results in more current flow through the surface of the antenna, which consequently increases the impedance bandwidth of the antenna, as depicted in Figure 3. From Figure 4, it can be seen that with each modification, the more current induced in the radiator causes the resonance at the selected frequency, thereby eventually increasing the bandwidth of the proposed antenna. The final antenna after optimization has a wide |S11| > −10 dB impedance bandwidth at 6.95 GHz ranging from 2.73–9.68 GHz, as illustrated in Figure 3.

### 2.3. Optimization and Design Procedure

**Figure 5:**
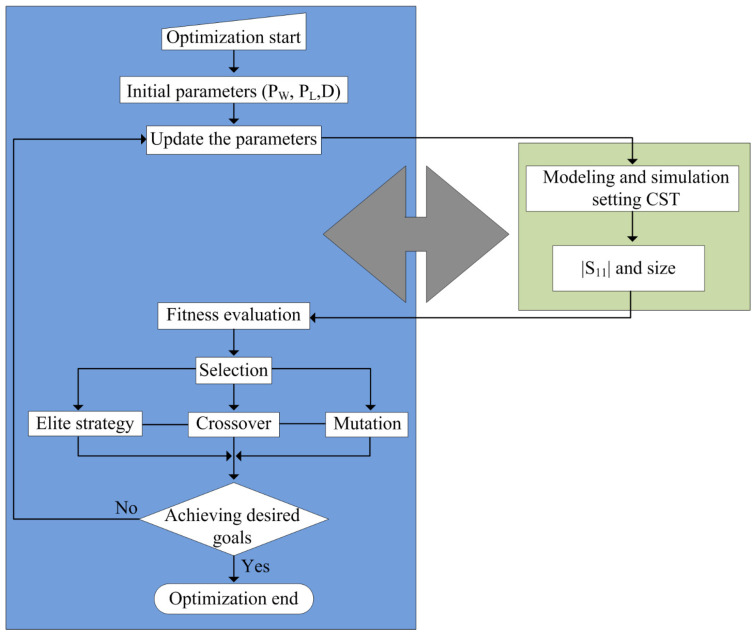
Flow chart explaining the optimization algorithm utilized for the proposed antenna.

The generic value of various parameters, including the length and width of the monopole antenna, along with CPW-feeding parameters as well as the impedance of the stub, can be estimated by various equations. However, due to the presence of various materials and connector losses, these values must be optimized to achieve the desired results. In the literature, various optimization techniques were used. However, due to its ease of use and lustiness, the CST Genetic Algorithm (GA) is utilized. Figure 5 depicts the working flow chart of the GA. The detailed description and working methodology of the GA are fully explained in [31].

**Figure 6:**
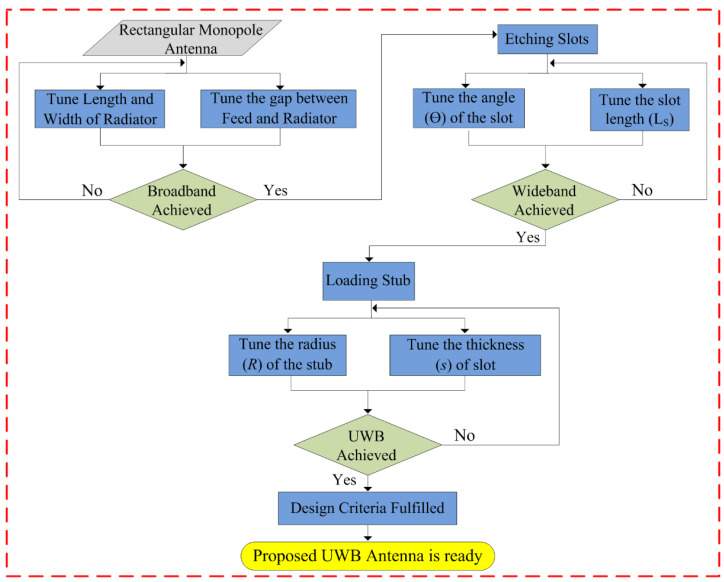
Flow chart explaining the optimization algorithm utilized for the proposed antenna.

The design methodology of the proposed flexible UWB antenna is shown in Figure 6. The antenna can be summarized as the following three steps:

*Step-1:* Initially, a quarter-wave monopole antenna is designed using the equations provided in the literature. Afterwards, the length, width, and gap between CPW ground and the radiator are optimized using GA to achieve the maximum possible bandwidth without affecting the overall size of the antenna.

*Step-2:* Later, the etching slot technique is utilized to achieve wideband behavior, where a simple structure is employed to avoid any structural complexity. Then, the length and angle of the slot are optimized to achieve wideband behavior. It is important to note that the length and width of the radiator, as well as the gap between the radiator and CPW ground, remain constant as of step 1.

*Step-3*: Finally, a shorted stub is loaded at the top edges of the antenna to improve the impedance matching of the antenna. The resultant antenna offers UWB behavior while covering almost 95% of the band spectrum allocated globally for UWB applications. In this step, the thickness and internal radius of the stub are optimized initially, along with a slight optimization of the angle and length of the slot.

By following the aforementioned steps 1, 2, and 3, a compact-sized antenna with incredibly low structural complexity as well as UWB bandwidth can be designed. A detailed theoretical analysis and mathematical equation validate the proposed work’s scientific contribution.

## 3. Performance Parameters of the Antenna

### 3.1. Reflection Coefficient

**Figure 7:**
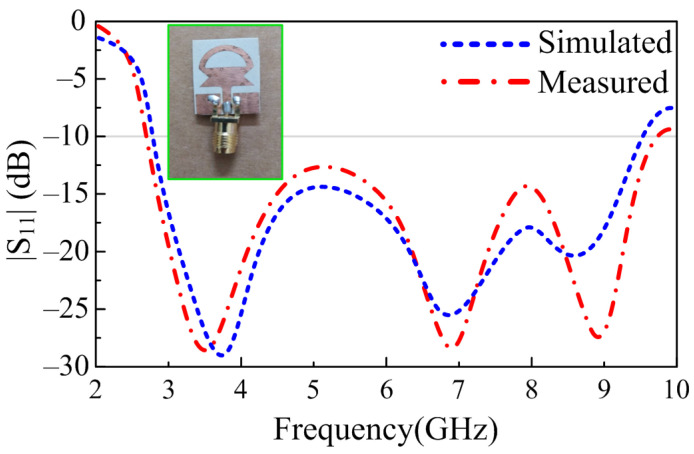
Comparison among the predicted and measured reflection coefficients of the proposed work.

In order to substantiate the theoretically proven results, a prototype of the antenna was fabricated using a standard chemical etching process. A gold-plated SMA connector having an impedance of 50 Ω is utilized for excitation of the antenna, as depicted in Figure 7. The reflection coefficient of the proposed UWB antenna is measured using an E5063A Vector Network Analyzer (VNA) by KEYSIGHT Technologies, with a maximum frequency range of up to 18 GHz. Figure 7 provides the comparison between theoretical and measured results; it can be observed that the measured results are in good agreement with the simulated results. The measured results offer an UWB of 7.05 GHz, which is 140% of the initial central frequency of 5 GHz. The proposed work, as shown in Figure 7, provides |S11| > −10 dB impedance bandwidth ranges from 2.70–9.75 GHz.

### 3.2. Conformal Analysis

**Figure 8:**
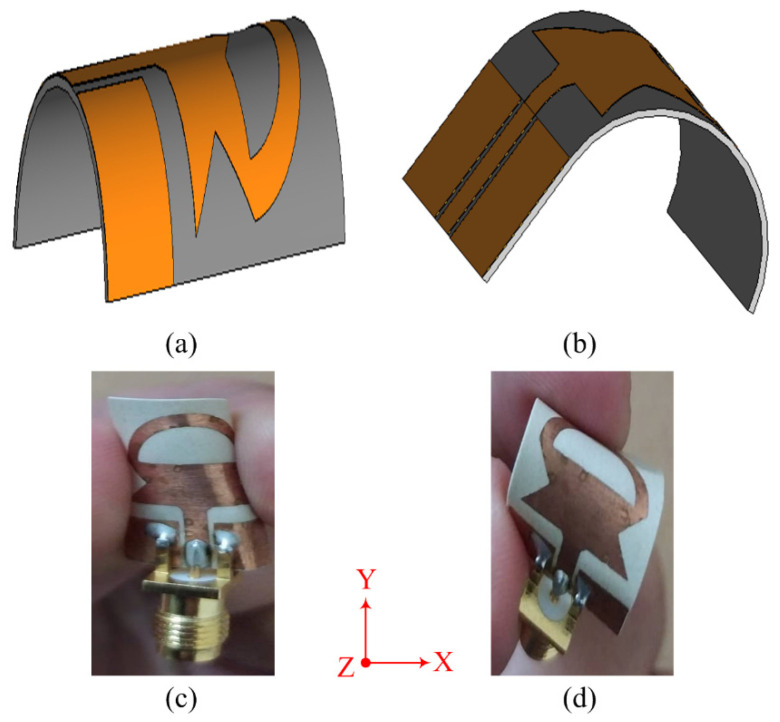
Simulation setup for conformal structure (**a**) *X*-axis (**b**) *Y*-axis; fabricated prototype of the proposed antenna bent along (**c**) *X*-axis (**d**) *Y*-axis.

**Figure 9:**
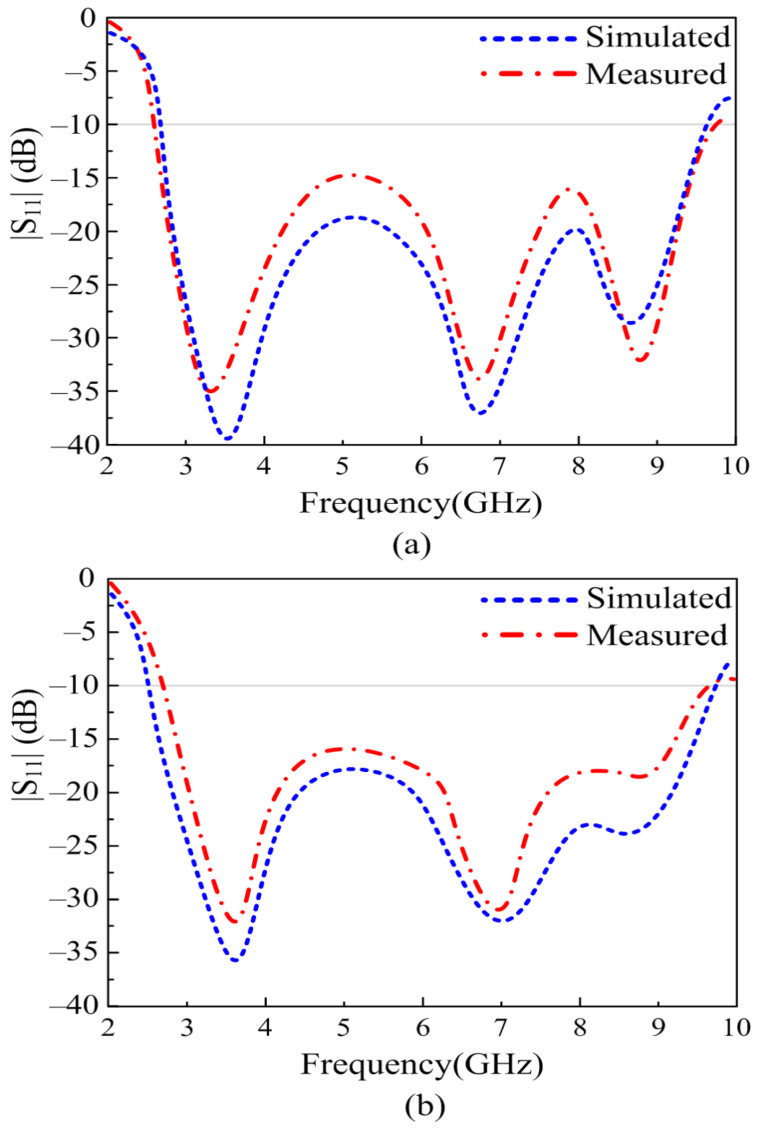
Comparison between predicted and measured reflection coefficients of the antenna under conformal conditions along the (**a**) *X*-axis and (**b**) *Y*-axis.

In working effectively with the flexible electronics, the antenna should offer a stable result in both conformal and non-conformal conditions. The antenna is bent in both the *X* and *Y* axes for this purpose, as shown in Figure 8 and Figure 9, respectively. The bending radius for both simulation and measurements is chosen to be 10 mm. Figure 9 depicts the comparison between the simulated and measured results of antennas under bending conditions. It can be observed from Figure 9a that when the antenna is bent along the *X*-axis, the return loss of the antenna gets further improved as compared to the antenna without any bending. Furthermore, the bandwidth remains identical to the rigid case. On the other hand, when the antenna is bent along the *Y*-axis, as shown in Figure 9b, a slight improvement in bandwidth as well as return loss is observed, similar to the results observed in non-conformal conditions. Thus, it can be concluded that the antenna offers almost identical results in both scenarios, which shows the performance stability of the proposed work. Furthermore, the strong agreement between simulated and measured results also highlights the performance stability of the antenna in terms of return loss.

### 3.3. Far-Field Analysis

#### 3.3.1. Measurement Setup

**Figure 10:**
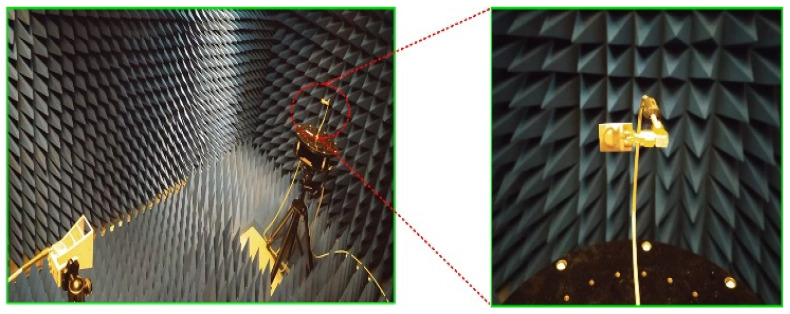
Measurement setup for far-field analysis of the propped flexible UWB antenna.

The fabricated prototype is further utilized to measure the far-field parameters of the proposed work. In order to this, the antenna was placed inside the anechoic chamber in front of the reference Horn antenna, which has a broadband of up to 12 GHz. The measurement setup, along with a close snap of the proposed work, is depicted in Figure 10.

#### 3.3.2. Radiation Pattern

**Figure 11:**
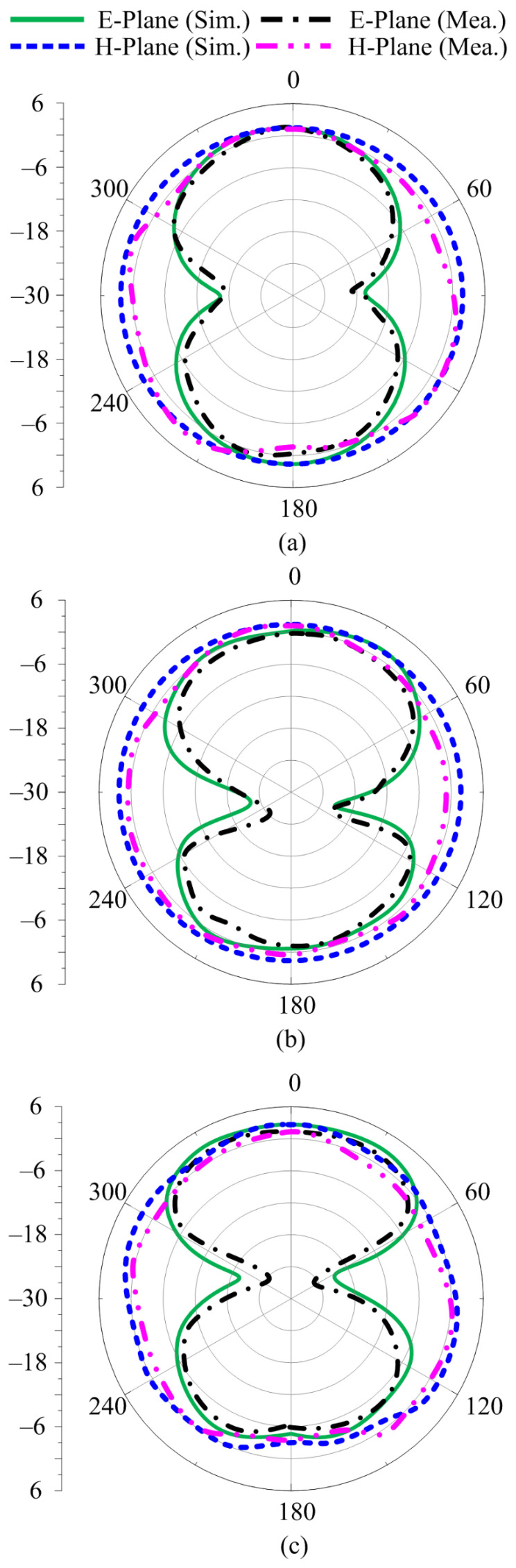
Comparison of predicted and measured radiation patterns at (**a**) 3.5 GHz, (**b**) 6.8 GHz, and (**c**) 9 GHz.

Figure 11a–c depict the radiation pattern of the proposed antenna at the selected frequencies of 3.5 GHz, 6.8 GHz, and 9 GHz, respectively. In all the selected frequencies, the antenna provides an omnidirectional radiation pattern in the h-plane, while in the e-plane, the antenna provides a monopole-like bidirectional radiation pattern. It can also be seen that as the frequency increases, the radiation pattern becomes slightly distorted, owing to the fact that the antenna size grows relatively large. Furthermore, the measured results offer a strong agreement with the simulated results over all frequencies, as depicted in Figure 11.

#### 3.3.3. Radiation Pattern under Conformability

**Figure 12:**
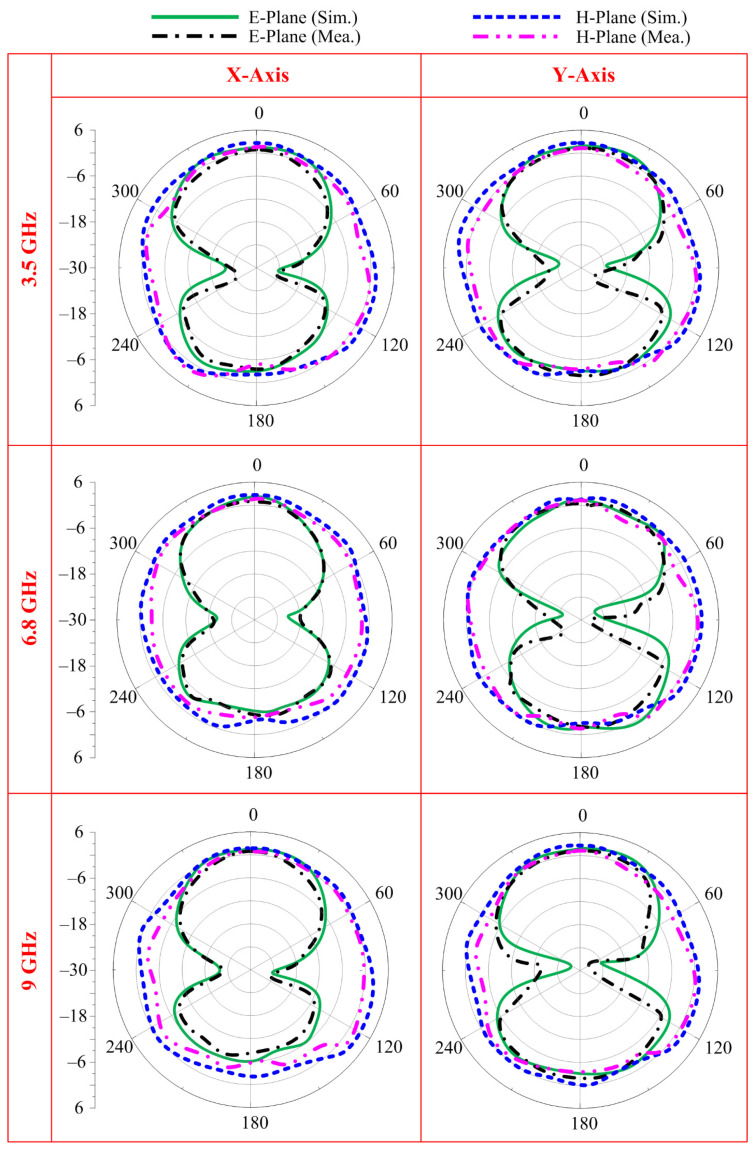
Comparison between predicted and measured radiation patterns of the proposed antenna.

The prototype is then used to measure the radiation pattern under bending conditions. The radiation is measured at the selected frequencies of 3.5 GHz, 6.8 GHz, and 9 GHz while bending the antenna along the *x*- and *y*-axes, as shown in Figure 12. It can be observed that at all selected frequencies, the antenna offers a nearly identical radiation pattern as compared to the antenna under normal conditions. A little deviation from the original pattern is due to conformability in the radiating structure. Moreover, the simulated and measured results also show strong agreement with each other, stating the performance stability of the proposed antenna in terms of radiation pattern.

#### 3.3.4. Gain of UWB Antenna

**Figure 13:**
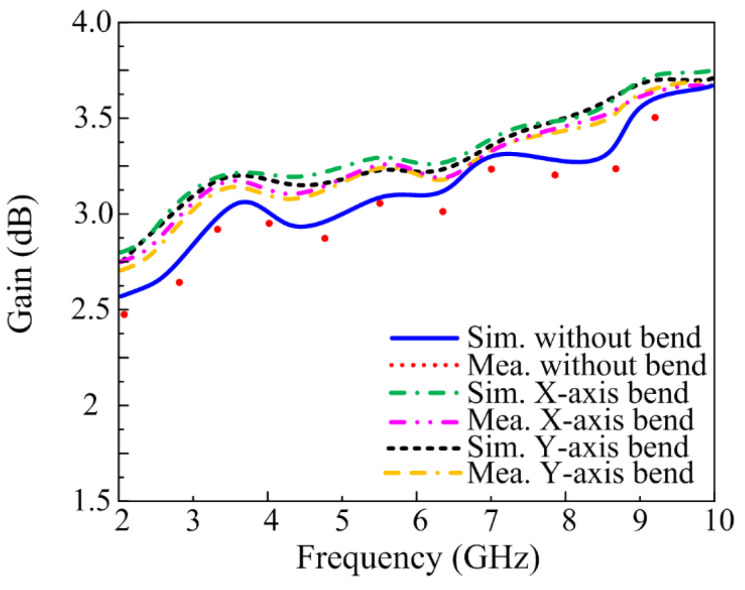
Gain of the proposed work for conformal and non-conformal scenarios.

The gain of the proposed antenna is also measured for both conformal and non-conformal conditions. It can be observed from Figure 13 that the antenna offers a gain of 2.5 dB in operational bandwidth. Moreover, it can also be observed that for conformal conditions, a slight increase in gain is achieved. It is due to a slight deviation in the radiation pattern. Furthermore, a strong agreement is observed between the theoretical and measured values of the gain.

### 3.4. Group Delay

The time-domain analyses are another crucial factor while designing the UWB antenna, as they are useful to understand the propagation through the antenna. The group delay of any UWB can be calculated by using change in phase with respect to change in frequency and can be estimated by using the following relation provided in [32].

(6) $$\mathrm{Group}\;\mathrm{Delay}=–\frac{1}{2\pi }\frac{d\theta }{df}$$

**Figure 14:**
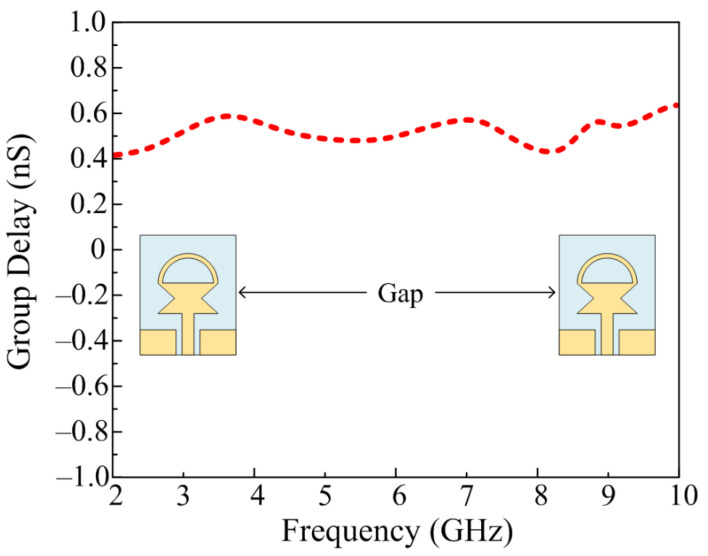
Group delay of the proposed UWB antenna.

In order to work efficiently, the UWB antenna should possess a constant group delay, ideally. However, due to the presence of various losses in the materials used for designing the antenna, a little deviation is acceptable. The proposed antenna offers a group delay range of 0.4–0.6 nS with an average value of 0.5 nS over the entire bandwidth, as shown in Figure 14.

### 3.5. Comparison with State-of-the-Art

Table 1 presents the comparison of the proposed antenna with the state-of-the-art. It can be observed from Table 1 that the antenna reported in [13,14,15,16,17,18,19,20,21,22] offers a bigger size as compared to the proposed work, while the antenna reported in [13,14,15,16,17,18,19] also offers structural complexity. Moreover, the antenna reported in [14,15,16,17,18,19,22] exhibits low gain as compared to the presented work. Therefore, it can be concluded that the proposed work over-performs the related work by offering a good combination of compact size, wideband, moderate gain, low structural complexity, and strong comparisons among simulated and measured results in both rigid and bending conditions.

## 4. Conclusions

This article presents the design of a compact-sized UWB antenna extracted from a conventional rectangular monopole by etching a pair of slots and a loading stub. The antenna is designed using ROGERS 5880, a flexible material with an extremely low dielectric loss of 0.0009, a thickness of 0.254 mm, and a dielectric constant of 2.2. Additionally, by means of two triangular slots along with a semi-circular-shaped short-ended stub, a narrow band antenna is converted to a UWB antenna without increasing the structural complexity. The antenna covers the band spectrum ranging from 2.73–9.68 GHz, has a minimum gain of >2.5 dBi, and has an omni-directional radiation pattern. Furthermore, when the antenna is bent along the *x* and *y* axes, its performance remains identical to that of the unbent antenna. Along with that, the strong comparison between simulated and measured results for both conformal and non-conformal scenarios shows the performance stability of the proposed UWB antenna. Moreover, the comparison with the state of the art shows that the presented work outperforms the related work. Thus, owing to the advantage of the CPW-feeding technique providing ease of integration with other electronics circuits and its size, compactness, wideband, and stable performance for both non-conforming and conforming scenarios, the proposed work is a strong potential candidate for wireless networks and small flexible as well as rigid devices requiring UWB antenna.

## Figures and Tables

**Table 1:** Comparison with State-of-the-Art.

Ref. no.	Size (mm^3^)	Bandwidth (GHz)	Gain (dB)	Structural Complexity	Flexibility
[13]	59.8 × 59.8 × 3.4	2–3	>2.5	High	No
[14]	39 × 39 × 1.6	2–13	>0.5	High	No
[15]	80 × 67 × 3.4	3.68–10.1	>0.9	High	Yes
[16]	28.1 × 17.1 × 1.4	5–14	>2	Moderate	No
[17]	40 × 22 × 0.1	2.5–11	>2	Moderate	Yes
[18]	33 × 22 × 0.125	4–10	>1	High	Yes
[19]	38 × 22 × 0.1	4–11	>0.3	Moderate	Yes
[20]	33.1 × 32.7 × 0.254	4–15	>2.5	Low	Yes
[21]	34 × 32.6 × 0.05	2–10	>2.8	Low	Yes
[22]	48 × 34.9 × 0.05	2–8	>−2.1	Low	Yes
Proposed Work	20 × 15 × 0.254	2.73–9.68	>2.5	Low	Yes

## Data Availability

Not applicable.
